# Chemical and Skincare Property Characterization of the Main Cocoa Byproducts: Extraction Optimization by RSM Approach for Development of Sustainable Ingredients

**DOI:** 10.3390/molecules26247429

**Published:** 2021-12-07

**Authors:** Catalina Agudelo, Karent Bravo, Ana Ramírez-Atehortúa, David Torres, Luis Carrillo-Hormaza, Edison Osorio

**Affiliations:** Grupo de Investigación en Sustancias Bioactivas, Facultad de Ciencias Farmacéuticas y Alimentarias, Universidad de Antioquia, Calle 70 No. 52-21, Medellín 50010, Colombia; catalina.agudelor@udea.edu.co (C.A.); karen.bravo@udea.edu.co (K.B.); anilla0122@gmail.com (A.R.-A.); david.torresb@udea.edu.co (D.T.); luis.carrillo@udea.edu.co (L.C.-H.)

**Keywords:** cocoa byproducts, antioxidant activity, antiaging properties, catechins, methylxanthines, cosmetic potential

## Abstract

Methylxanthines and polyphenols from cocoa byproducts should be considered for their application in the development of functional ingredients for food, cosmetic and pharmaceutical formulations. Different cocoa byproducts were analyzed for their chemical contents, and skincare properties were measured by antioxidant assays and anti-skin aging activity. Musty cocoa beans (MC) and second-quality cocoa beans (SQ) extracts showed the highest polyphenol contents and antioxidant capacities. In the collagenase and elastase inhibition study, the highest effect was observed for the SQ extract with 86 inhibition and 36% inhibition, respectively. Among cocoa byproducts, the contents of catechin and epicatechin were higher in the SQ extract, with 18.15 mg/100 g of sample and 229.8 mg/100 g of sample, respectively. Cocoa bean shells (BS) constitute the main byproduct due to their methylxanthine content (1085 mg of theobromine and 267 mg of caffeine/100 g of sample). Using BS, various influencing factors in the extraction process were investigated by response surface methodology (RSM), before scaling up separations. The extraction process developed under optimized conditions allows us to obtain almost 2 g/min and 0.2 g/min of total methylxanthines and epicatechin, respectively. In this way, this work contributes to the sustainability and valorization of the cocoa production chain.

## 1. Introduction

Vegetal residual biomass consists of residues produced in the field as a consequence of farming activities (agricultural or forestry) and is mainly a byproduct of the food and beverage industry. This biomass is utilized as fodder in animal feed, as landfill material and fertilizer, and is burned as fuel for energy production or simply disposed of as waste. These practices cause several environmental and sanitary issues. Residual biomass, which is one of the largest problems of the agro-industry today, generates millions of tons a year [1]. These residues are mainly derived from edible oils, fruit beverages, sugar, coffee, cereals (rice and wheat), and cocoa industries [2]. Therefore, it is important and necessary to integrate it into circular economic processes to reduce the negative environmental impact of the agro-industry and to contribute to food waste valorization. In addition to lignocellulosic residues, residual biomass is an important source of bioactive compounds, such as carotenoids, peptides, pigments, and polyphenols, which have functional properties and can be used as functional ingredients in many industries, giving high value to products.

Cocoa (*Theobroma cacao* L.) is a Latin American origin plant; however, currently, it is widely cultivated in many tropical regions around the world. According to reports, 4.84 million tons of cocoa were produced worldwide during 2020 [3]. Countries such as Brazil, Cameroon, Ivory Coast, Ecuador, Ghana, Indonesia, and Nigeria are the main producers [4]. The cocoa product market earned $24.5 billion in 2019 and is projected to have an estimated value of $30.2 billion in 2026 [5]. For example, in Colombia, a growing cocoa country, the production of this crop in 2018 was 101,099.94 tons and is expected to increase to 160,000 tons in the future [6]. Currently, chocolate and cocoa-derivative foods are obtained from cocoa beans after field and industrial processing. However, during processing in production units, approximately 80% of cocoa fruit is discarded as byproducts [7]. Therefore, a vast amount of waste and byproducts, such as cocoa pod husks (PH), flat cocoa beans or “pasilla” (PA), musty cocoa beans (MC), second quality cocoa beans (SQ), cocoa sweating, and cocoa bean shells (BS), are obtained and represent for producing countries an important environmental concern. The PH is the exocarp, the outer part of the fruit. PA is the set of small and lean beans with a maximum almond content of 40–60% [8]. The overripe beans and those that are attacked by birds and squirrels are considered SQ. BS is the surrounding layer of cocoa beans that is removed from the cotyledons (nibs) to allow homogeneous roasting in the process of generating cocoa products [2]. Additionally, the elimination of these byproducts is done without a suitable treatment, which also generates putrid odors and plant diseases for producing countries [2].

Some studies have analyzed the use of cocoa byproducts in the development of functional ingredients for food, cosmetic and pharmaceutical formulations. Thus, compounds such as pectin, methylxanthines, and mainly dietary fiber and flavanol-type polyphenols have been reported. In this sense, PH is recognized for its high content of dietary fiber and pectin, BS is composed of a lignincellulosic complex particularly rich in dietary fiber, and cocoa sweating has rich composition of sugars and minerals [2,4]. Antioxidant, anti-inflammatory, preventive against cancer, cardiovascular and diabetes diseases, and beneficial intestinal properties have been reported for flavanols, and in general, for cocoa phenolic compounds [9,10,11,12]. Cocoa-derived phytochemicals have also been effective as in vitro and in vivo approaches for skincare. For example, ultraviolet B (UVB) produces skin damage, and catechin protects against these negative effects by modulating antioxidant enzyme activities [13]. Cocoa pod extract inhibited collagenase, elastase and tyrosinase, skin aging-associated enzymes [14], reduced wrinkles, and increased skin hydration in human volunteers [15]. On the other hand, methylxanthin-type alkaloids, mainly caffeine and theobromine, are abundantly present in cocoa and its byproducts, and different pharmacological properties have been attributed to these compounds. Their effects include protective actions on the respiratory and cardiac systems as well as on skeletal muscle, and stimulation of the central nervous system (CNS) [16]. In addition, in the brain, methylxanthines stimulate serotonin release, interact with the adenosine receptor and activate the dopamine receptor which participates in the regulation of various functions such as motor behavior, emotion, and affectivity [17]. Therefore, both types of metabolites, methylxanthines, and polyphenols, should be jointly considered in a cocoa waste management strategy for their exploitation, which could be extracted and used conveniently due to its biological properties. In this regard, studies have not quantitatively analyzed some polyphenol groups and methylxanthines in the different byproducts, accompanied by anti-aging skin tests, and compared with high-quality cocoa beans.

Developing a sustainable process depends on many factors, the source is very important, but the selection of a green, efficient, and scalable extraction process is mandatory. The high-intensity ultrasound extraction in continuous flow mode is a novel and scalable configuration that concentrate all the energy in a small reactor while the solvent with the plant material suspended recirculates through it. Under this configuration, the extraction productivity has increased more than three-fold in comparison with ultrasound bath in biflavonoid extraction from *Garcinia madruno* [18]. Therefore, it is a very attractive technology to be used in the valorization of agro-industrial by-products as those generated in the chocolate industry.

This study presents a quantitative analysis of cocoa flavanols and methylxanthines and skincare properties measured by antioxidant assays and inhibition of skin aging-associated enzymes, in various cocoa byproducts compared to premium and ordinary raw materials. A further objective was to optimize the extraction and enrichment of methylxanthines and flavanols from cocoa byproduct extracts using response surface methodology (RSM) applying high-intensity ultrasound in continuous flow mode to add value to cocoa byproducts as a possible source of ingredients of industrial interest.

## 2. Results

### 2.1. TPC and Antioxidant Activity in Cocoa Byproducts

**Figure 1:**
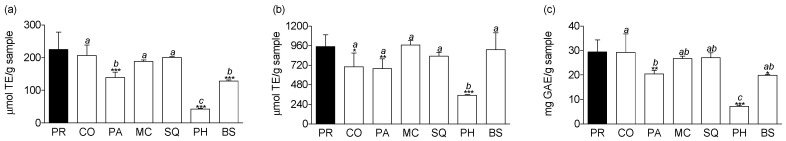
TPC and antioxidant activity and skin protective properties of cocoa samples. (**a**) FRAP-value; (**b**) ORAC-value; (**c**) Total phenolic content. BS: cocoa bean shell; CO: ordinary cocoa bean; KA: Kojic acid; MC: musty cocoa bean; OA: oleanolic acid; PA: pasilla; PH: cocoa pod husk; PR: premium cocoa bean; and SQ: second quality cocoa bean. Different letters indicate significant differences (*p* < 0.05) between cocoa samples (Bonferroni’s multiple comparison test). For each cocoa sample, asterisk denotes significantly difference in comparison with the control PR (Dunnett’s multiple comparison test; * *p* < 0.05, ** *p* < 0.01 and *** *p* < 0.001).

Five types of cocoa byproducts were investigated to their antioxidant effects determined by ORAC and FRAP assays. The TPC value was determined using Folin Ciocalteu’s method (Figure 1). The byproduct effects were compared with those of premium cocoa beans (PR), a cocoa of high quality. MC and SQ byproducts showed the highest phenol contents and antioxidant activity. These extracts showed activities similar to those of the reference sample. For example, MC extract showed a TPC value of 27.05 mg GAE/g and FRAP and ORAC values of 200.45 and 969.82 μmol TE/g, respectively. The BS extract stood out for its high antioxidant activity according to the ORAC assay. PA and PH showed low values compared to the PR extract and to the other cocoa byproduct extracts. PH is the residue with the fewest properties, with a TPC content of 7.17 mg GAE/g and an ORAC value of 351.60 μmol TE/g and FRAP value of 42.33 μmol TE/g.

### 2.2. Quantification of Catechins and Methylxanthines by HPLC-DAD-FLD in Cocoa Byproducts

PR extract was employed as a reference control. The catechin content was higher in the SQ extract with 18.15 mg/100 g of sample than in the PR extract with 17.21 mg/100 g of sample (Table 1). The CO extract and SQ extract showed high contents of epicatechin, with 279.5 mg/100 g of sample and 229.8 mg/100 g of sample, respectively. PR extract had the highest value with 383.1 mg/100 g of sample. Catechin and epicatechin were not found in the PH extract. Cocoa byproducts were also analyzed by HPLC-DAD-FLD for their quantification of methylxanthines. In general, the content of caffeine was lower (*p* > 0.05) than the theobromine content. The determined caffeine concentration varied between 9 and 267 mg/100 g of sample, whereas the theobromine content varied between 38 and 1085 mg/100 g of sample (Table 1). The theobromine content was higher in cocoa byproducts, such as BS and MC extracts, than in the PR extract (834.7 ± 29.8 mg/100 g of sample). The BS extract showed the maximum amount of this compound with 1085 mg/100 g of sample (*p* < 0.001). BS and PA extracts had the highest caffeine contents, 267.0 and 147.6 mg/100 g of sample, respectively. In the PR extract, caffeine was quantified as 141.7 mg/100 g of sample.

### 2.3. Anti-Aging Effects of Cocoa Byproducts

**Figure 2:**
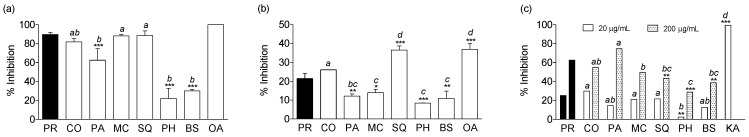
Skin protective properties of cocoa samples. (**a**) Percentages of inhibition for collagenase; (**b**) Percentages of inhibition for elastase; (**c**) Percentages of inhibition for tyrosinase. In the enzyme inhibition, the values are expressed as the mean ± SD; BS: cocoa bean shell; CO: ordinary cocoa bean; KA: Kojic acid; MC: musty cocoa bean; OA: oleanolic acid; PA: pasilla; PH: cocoa pod husk; PR: premium cocoa bean; and SQ: second quality cocoa bean. Different letters indicate significant differences (*p* < 0.05) between cocoa samples (Bonferroni’s multiple comparison test). For each cocoa sample, asterisk denotes significantly difference in comparison with the control PR (Dunnett’s multiple comparison test; * *p* < 0.05, ** *p* < 0.01 and *** *p* < 0.001).

In this study, collagenase and elastase inhibitory activity were evaluated using oleanolic acid (250 µM) as an assay positive control, and PR extract was used as a reference control to compare with cocoa byproduct extracts. In the collagenase enzyme (Figure 2a), the SQ, MC, and CO extracts produced a strong effect with 89%, 88%, and 82% inhibition, respectively, whereas the PH extract had the lowest value with 22% inhibition. In the elastase enzyme (Figure 2b), the highest inhibition was also observed for the SQ extract with 36%. In comparison with the reference control (21% inhibition), the SQ extract produced a statistically significant (*p* < 0.001) effect under the evaluated conditions. Similar to a previous test, the PH extract had the lowest value with 8% inhibition. In this work, two extract concentrations were also evaluated (20 μg/mL and 200 μg/mL) for tyrosinase activity using kojic acid as the assay positive control and PR extract as the reference control (Figure 2c). PA extract showed a strong inhibitory effect with 75%, followed by CO and MC extracts with 55% and 49%, respectively. In comparison with the reference control, these byproduct extracts did not show significant differences at either concentration evaluated. SQ with 43%, BS with 39%, and PH with 29% inhibition were the cocoa byproduct extracts with the lowest activity evaluated at 200 μg/mL. The extracts evaluated at 20 μg/mL showed similar behavior except for CO and MC extracts, which presented the highest activity with 30% and 21% inhibition of tyrosinase enzyme, respectively.

### 2.4. Optimal Extraction Processing Parameters Using Response Surface Methodology (RSM) in Cocoa Byproducts

To obtain functional ingredients applying high-intensity ultrasound extraction, BS was selected and various influencing factors in the extraction process were investigated using response surface methodology (RSM). The extraction time (*X*_1_), amplitude (*X*_2_), and solid/solvent concentration (*X*_3_) were the optimized independent variables with the application of the Box–Behnken experimental design under the RSM methodology. Table 2 displayed the response of ANOVA for all runs. The result showed that the effects of the independent variables *X*_1_ and *X*_3_ were significant with a level of *p* < 0.05. The other variable was observed to be not significant with a level of *p* > 0.05. Based on results on responses, a second-order polynomial model was established, and the predicted values were obtained by fitting the experimental data of extraction in the model. Table 3 shows the experimental and predicted values along with independent variables for each point of the experimental design. In all cases, the square coefficients of the correlation value (R^2^) exceeded 0.8, indicating a high correlation between the observed value and the predicted value, and in general, a reasonable performance of the model.

## 3. Discussion

Cocoa is a source of polyphenols and methylxanthines, and the attention in this study has been directed to the analyses of cocoa byproducts in the production of functional ingredients with high value for food, cosmetic and pharmaceutical formulations. Previously, a rapid method for the joint determination of catechins and methylxanthines by using HPLC-DAD-FLD in food matrices derived from cocoa was implemented [19]. Here, the analysis was carried out on the main cocoa byproducts. Thus, five byproducts were studied with the optimized and validated method, and the results were compared with the extracts of two commercial cocoa samples. Through the chemical and biological characterization of extracts from cocoa agro-industrial wastes by different techniques, the use of these byproducts as a potential source of ingredients for the pharmaceutical sector was verified.

In relation to the antioxidant activity of cocoa byproducts, the antioxidant capacity determined by the FRAP assay was less than one-third of the ORAC values. Probably the difference is based on the different chemical basis of both methods. The first is related to an electron transfer mechanism, while the second is a method that is based on the transfer of hydrogen atoms [20]. In addition, the correlation analysis between TPC and antioxidant capacity was conducted (see Supplementary Material). TPC and FRAP showed a strong correlation (*p* < 0.05). The coefficient of correlation was 0.960, suggesting that antioxidant properties measured through a reduction mechanism are mainly related to polyphenols. The intermediate correlation coefficient of 0.780 determined for TPC and ORAC values, suggests that other compounds may contribute to the antioxidant activity in addition to polyphenols. Cocoa polyphenols have been reported as highly antioxidant compounds [9,10,11,12]. Thus, it is not uncommon to think that polyphenols may be associated with the antioxidant effects of cocoa byproducts. However, studies have not quantitatively analyzed some polyphenol groups in the different cocoa byproducts [4,21]. In this regard, catechin, epicatechin, and procyanidins represent the main proportion of cocoa polyphenols [22]. The molecular structures of catechin and epicatechin are similar to those of other polyphenols, such as epigallocatechin and procyanidin oligomers and polymers. Therefore, the adsorption properties would also be similar [23]. Nevertheless, the favorable pharmacological effects of cocoa have been related to the flavonoid content, particularly flavanols. For example, antioxidant and anti-inflammatory activities have been shown for epicatechin [24,25]. Therefore, flavanol quantification in a cocoa byproduct with the HPLC-DAD-FLD method was determined in addition to TPC. The results are shown in Table 2. In general, the epicatechin concentration was quite higher than the catechin concentration (*p* > 0.05) in cocoa byproducts. These values are consistent with previous reports, which show that epicatechin is the main flavanol in cocoa [26]. In addition, SQ is constituted as the main byproduct due to its catechin content.

Cocoa byproducts were also analyzed by HPLC-DAD-FLD for their quantification of methylxanthines. These alkaloids represent the most important stimulant compounds in cocoa-derived products, with caffeine and theobromine being the representative alkaloids [22]. The BS cocoa byproducts showed the maximum amount of theobromine with 1085 mg/100 g of sample (*p* < 0.001). Studies in which the cocoa shells have been analyzed have shown that this byproduct contains compounds such as caffeine, theophylline and mainly theobromine. The high content of methylxanthines, may be due to a consequence of migration processes from the grain to the shell during processing [4]. In this way, BS is constituted as the main byproduct due to its methylxanthine content. The biological properties of methylxanthines could favor the pharmaceutical use of cocoa byproducts. Different properties have been reported, e.g., stimulation of bronchodilation, diuresis, gastric secretion, and CNS [27]. The biological effects of methylxanthines contribute to obesity, nervous, respiratory, cardiac and renal system disorder management, and fertility problems [28]. Additionally, theobromine is an activator of the CNS and is employed as an active principle for bronchial relaxation and asthma control [29]. Theobromine has coronary vasodilator, diuretic, and smooth muscle relaxant properties [30]. In addition, small amounts of theobromine increase serotonin release in specific regions of the CNS (cerebellum and cerebral cortex) [17]. This effect can suggest that theobromine could be used in the development of products to control emotions and mood. At the cosmetic level, theobromine has been proposed as an alternative to fluoride to protect teeth from decay [31], and in the Cosmetic Ingredient list of the European Commission-CosIng, it is accepted as a cosmetic ingredient with the functions of skin conditioning and fragrance [32].

The degradation and breakdown of elastin and collagen fibers, as well as different alterations of the dermal connective tissue, are facts that characterize skin aging; with oxidative processes, skin aging has a central role and is considered to be the cause of the appearance of skin laxity, deep wrinkles and telangiectasias [33]. Biochemically, this process is initiated by the action of matrix metalloproteinases (MMP-1), which correspond to zinc-dependent endopeptidases that proteolytically degrade the proteins of the skin matrix. Among endopeptidases, the main action of the collagenase and elastase enzymes can be observed. The first is responsible for the partitioning of collagen, a fundamental component of the extracellular matrix that provides the stability of the skin. The second enzyme degrades elastin fibers, a protein responsible for the elasticity of the skin, located mainly in the connective tissues [33]. Thus, the inhibition of both proteolytic enzymes could constitute an antiaging target to be analyzed in cocoa byproducts in in vitro models. In this sense, obtaining bioactive extracts from cocoa byproducts to protect the components of the extracellular matrix represents an achievement in the development of natural ingredients with antiaging effects. In this regard, it has been reported that catechins provide beneficial effects on skin health and protection against ultraviolet (UV) radiation and pollution due to their scavenging free radical activity and reduced degradation of extracellular matrix components [34]. Additionally, these compounds promote collagen synthesis and inhibit matrix metalloproteinase enzyme production [35]. A study has shown that epicatechin attenuates the oxidative damage in dermal fibroblasts caused by UVA radiation and suggests that epicatechin protects against deleterious effects of sunlight [36]. On the other hand, tyrosonase inhibition was also analyzed, as it is a key enzyme in melanogenesis, and as a strategy in the search and development of skin whiteners [37]. Some tyrosinase inhibitors such as arbutin and hydroquinone are used in cosmetic products. Nevertheless, limitations in terms of low activity, skin penetration and toxicity, leads to search for effective, safe and specific tyrosinase inhibitors [37]. The correlation analysis between TPC and inhibitory activity of enzymes was conducted for all extracts evaluated (see Supplementary Material). TPC and anti-tyrosinase activity showed a strong correlation, followed by TPC and anti-collagenase activity (correlation coefficients greater than 0.888). These results suggest that polyphenols have an important role in the inhibition of the mentioned enzymes. The intermediate correlation coefficient of 0.670 determined for TPC and anti-elastase activity, suggests that other compounds may contribute to the enzymatic inhibition in addition to polyphenols.

**Figure 3:**
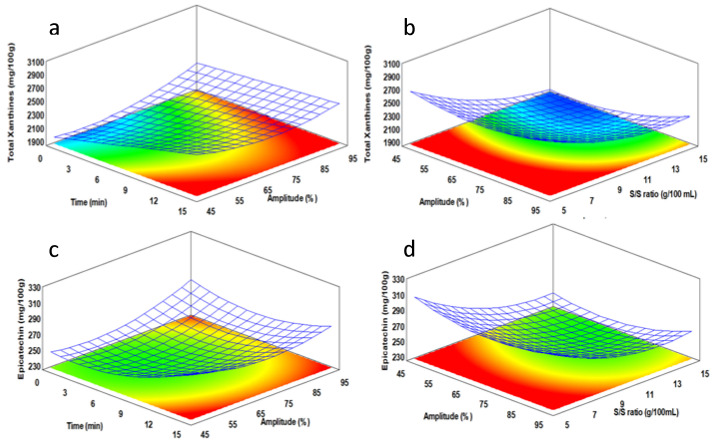
3D response surface plots. Combined effects of the high-intensity ultrasound extraction conditions, on the recovery of (**a**,**b**) total xanthines (caffeine + theobromine); and (**c**,**d**) epicatechin.

In the extraction process study by RSM, BS was selected over the other byproducts because it has the highest value of methylxanthines, presents a medium concentration of epicatechin, and has excellent availability and manageability as an agro-industrial residue. The response surface plots obtained from mathematical models were obtained for understanding the relationship between the independent variables (Figure 3). Both responses showed a similar profile. A time effect was observed when amplitudes between 45 and 75 (%) were applied. The content of phytochemicals increases proportionally with an increase in the extraction time under these conditions. When the maximum amplitude was applied (95%), there was no significant impact on the compounds extracted with time, and the maximum values of total methylxanthines and epicatechin extracted were obtained after 5 min of extraction (Figure 3a,c). Thus, the results confirm that the increase in the amplitude of the extraction system, which is an increment of the total energy and cavitation in the liquid phase, is related more to a kinetic effect on the optimization of extraction time required, i.e., the amplitude improves the productivity of the total amount of extracted compounds. This finding is consistent with other reports of phytochemical extraction when a dynamic high-intensity extraction process is performed [18]. On the other hand, the solid/solvent concentration was the most significant variable to improve the extractability of xanthine and epicatechin by the amount of cocoa shell used. The best results were observed at smaller solid/solvent concentrations. The optimal range would be between 5 and 9 g of plant material by 100 mL of solvent (Figure 3b,d). Dynamic high-intensity extraction is based on a recirculation process of the solid-liquid dispersion by a reaction chamber, where cavitation is focalized [18]. Therefore, decreasing the amount of solids improved the fluidization of the system and increased the mass transfer rate by increasing the movement of particles and the focalization of cavitation in a smaller number of particles to extract.

The application of regression equations allowed analysis of extraction conditions. The theoretical maximum values and optimal conditions for each response were total xanthines content (y = 2898.44 mg/100 g; *X*_1_ = 15 min, *X*_2_ = 94.8% and *X*_3_ = 5 g/100 mL) and epicatechin (y = 330.96 mg/100 g; *X*_1_ = 15 min, *X*_2_ = 94.9% and *X*_3_ = 5 g/100 mL). For both responses, the optimal variables obtained were practically the same. Although the “optimal” extraction time was 15 min with 95% amplitude, the analysis of the response surfaces under these conditions indicates that it is not necessary all this time. In fact, there was no significant difference between 5 and 15 min. Therefore, to avoid excessive energy consumption and promote the potential productivity of this process, the extraction time should be fixed at 5 min to maintain the values obtained for the other variables. The extraction process developed under optimized conditions would obtain almost 2 g and 0.2 g of total xanthines and epicatechin per minute, respectively. This result suggests that after applying some concentration and purification steps, it is feasible to develop functional ingredients based on xanthine and epicatechin from BS by employing dynamic high-intensity ultrasound extraction.

## 4. Materials and Methods

### 4.1. Reagents

(±)-6-Hydroxy-2,5,7,8-tetramethylchromane-2-carboxylic acid (Trolox), 2,2-azobis (2-methylpropionamidine) dihydrochloride (AAPH), 2,4,6-Tris (2-pyridyl)-s-triazine (TPTZ), fluorescein sodium salt, Folin–Ciocalteu reagent, gallic acid, dimethyl sulfoxide (DMSO) and the mushroom tyrosinase and L-tyrosine were purchased from Sigma-Aldrich (St. Louis, MO, USA). Acetic acid (CH_3_COOH) was purchased from Mallinckrodt Pharmaceuticals (Chesterfield, Derbyshire, England). Ferric chloride (FeCl_3_), HPLC grade solvents (acetonitrile, ethanol and hydrochloric acid (HCl)) and sodium carbonate were purchased from Merck KGaA (Darmstadt, Hesse, Germany). KH_2_PO_4_, NaC_2_H_3_O_2_ and Na_2_HPO_4_ were purchased from Carlo Erba Reagents (Sabadell, Spain). The assay kits EnzChek^®^ Elastase and EnzChek^®^ Gelatinase/Collagenase were obtained from Molecular Probes Inc. (Eugene, OR, USA).

### 4.2. Samples and Extraction of Plant Material

Samples were obtained from cocoa-producing farms of the Urabá Antioqueño region (Apartadó and Chigorodó). Fermented and sun-dried cocoa beans were employed. Cocoa byproducts were classified as cocoa pod husks (PH), flat cocoa beans or “pasilla” (PA), musty cocoa beans (MC), second quality cocoa beans (SQ) and cocoa bean shells (BS). Premium cocoa beans (PR) were used as reference controls because they are healthy beans without any infection or physical damage. Ordinary cocoa beans (CO) are commercial cocoa and were also used to compare results. PR are characterized by a minimum weight of 120 g/100 beans, and CO are characterized by a minimum weight of 105–119 g/100 beans [8]. Fresh material was dried at 40 °C for 5 days. Once all samples were dry, the material was mechanically ground until a homogeneous particle size is obtained. Later, the samples were saved at −20 °C protected from moisture and light.

The samples were subjected to ultrasonic-assisted extraction twice using the methodology previously described [38] with some modifications. Around 100 mg of the samples were degreased with hexane (2 mL), extracted in an ultrasonic bath (Elma P60H, Singer, Germany) at a fixed power of 700 W, for 8 min and centrifuged for 15 min at 13,000× *g* rpm. The fat-reduced samples were dried under reduced pressure and then extracted at room temperature with 2 mL of aqueous ethanol (70%) for 50 min in the same ultrasonic equipment at 30 ± 4 °C. After the process, the extracts were centrifuged at 13,000× *g* rpm for 20 min at 4 °C. Around 2 mL of the supernatant were withdrawn into a volumetric flask, which were protected from the light and stored at −20 °C until use.

### 4.3. Analysis of Catechins and Methylxanthines

HPLC analyses were performed according to a method previously developed in our laboratory [19]. A Liquid Chromatography system of the 1200 Series (Agilent Technologies, Santa Clara, CA, USA). Additionally, a fluorescence detector (FLD) set at 280 and 230 nm excitation wavelengths and 320 nm emission wavelengths were employed. A column chromatography with 50 × 4.6 mm × 1.8 µm (Zorbax SB-C18 RRTT) was used for chromatographic separation at 35 °C. The samples were diluted in a mobile phase with a gradient consisting of acetic acid 0.1% (solvent A) and acetonitrile (solvent B). The gradient program profile was a mix of solvents: 0–1 min 95% A; 2–11 min 85% A; 12–15 min 75% A; and 16–18.5 min 95% A. The compounds caffeine, catechin, epicatechin and theobromine were identified by comparing the chromatographic properties to those of standards. All analyses were reproduced three times.

### 4.4. Total Phenol Content (TPC) and Antioxidant Activity

The TPC was determined through the Folin–Ciocalteu method that was previously applied [20,33]. For this, extract solution and distilled water (25 μL) was added to a 96-well plate. Next, Folin–Ciocalteu reagent and distilled water in relation 1:10 (125 μL) were added and mixed. Sodium carbonate (7.5% *p*/*v)* (100 μL) was added to each well, and after 60 min at 27 °C, the absorbance was read at 765 nm using a Synergy HT Multimode microplate reader (Biotek Instruments, Inc; Winooski, VT, USA). Gallic acid was used as standard, and from this, the quantifications of TPC were calculated. The results are expressed as milligrams of gallic acid equivalents per gram of extract (mg GAE/g).

The antioxidant activity was determined through two methods. The Oxygen Radical Absorbance Capacity (ORAC) assay and Ferric Reducing Antioxidant Power (FRAP) assay. The first was a method previously adapted in our laboratory [37]. For this, AAPH was used as peroxyl radical generator, fluorescein was employed as a fluorescent probe and Trolox was utilized as a standard in the assay. AAPH, fluorescein and samples were arranged in phosphate buffer at 92.4 mM (pH 7.4). Later, Trolox standard or sample solution (50 μL) was combined with fluorescein at 1.6 μM (150 μL), incubated for 30 min at 37 °C, and AAPH solution at 125 mM (50 μL) was added. Then, 60 measurements of the fluorescence were made during 120 min at excitation wavelength and emission wavelength of 485 nm and 520 nm, respectively. The ORAC values in relative terms were calculated and showed as μmol Trolox equivalent per gram of extract (μmol TE/g). The FRAP values of the samples were determined with a method also previously adapted in our laboratory [20,33]. The FRAP reducing solution included acetate buffer at 300 mM (pH 3.6), FeCl_3_·6H_2_O at 20 mM and TPTZ at 10 mM in HCl solution at 40 mM, at a 10:1:1 ratio. Trolox was employed as a standard. Initially, sample or Trolox (10 μL) was added to each well in a 96-well plate, combined with reducing solution (250 μL) and incubated in the dark at 37 °C for 10 min. Then, the absorbance was measured at 593 nm. The results were expressed as μmol Trolox equivalent per gram of extract (μmol TE/g).

### 4.5. In Vitro Anti-Aging Properties Determination-Enzyme Inhibition Activities

Collagenase inhibitory effects were calculated employing the EnzChek^®^ Gelatinase/Collagenase assay kit, according to the manufacturer’s specifications and following some modifications previously proposed [37]. The enzyme assay was performed with 5.0 µg/mL of the extracts. Oleanolic acid was used as a positive control at 250 µM. The fluorescence was measured at excitation wavelengths of 485 nm and emission wavelength of 515 nm, and the results were expressed as the inhibition percentage of collagenase. Additionally, elastase inhibitory effects were calculated using the EnzChek^®^ Elastase assay kit, according to the manufacturer’s specifications and following some previously proposed modifications [37]. The enzyme inhibitory assay was performed with 200 µg/mL of the extracts, and the positive control was oleanolic acid applied at 500 μM.

Anti-tyrosinase activity was performed with a method previously adapted [37], where kojic acid (100 μM) was employed as a reference inhibitor and the extracts were evaluated at 20 and 200 µg/mL. The absorbance was determined each minute for 20 min at 480 nm. The results are shown as the inhibition percentage of tyrosinase.

### 4.6. High-Intensity Ultrasound-Assisted Extraction Procedure for RSM

In all experimental runs, BS byproduct was extracted in an ultrasonic liquid processor LSP-500 (Sonomechanics Company, New York, NY, USA), equipped with an air-cooled piezoelectric transducer (ACT-500), a reactor chamber (304 stainless steel), a full-wave Barbell Horn™ (FBH, 21 mm tip diameter), and an ultrasonic generator of 500 W. For a continuous flow, the ultrasonic system was conditioned. The experiments were carried out in a batch size of 350 mL of solvent (50:50 water-ethanol), at a constant frequency of 20 kHz (±0.5), with agitation at 500 rpm and a cooling temperature of 15 °C. After multiple extractions, the mixture was centrifuged at 13,000× *g* rpm for 5 min at 4 °C. The pellet was washed with an additional 500 µL of extraction solution, and the supernatants were transferred to a volumetric flask. The volume was adjusted to 2.0 mL with water. The final extract was subsequently filtered through a 0.45-μm nylon membrane and stored at −20 °C in the dark until HPLC analysis.

A Box-Behnken experimental design (BBD) was made with 15-run 3^3^ to calculate the optimal ultrasound-assisted extraction (UAE) parameters for the extraction of the main methylxanthines and epicatechin from cocoa shell. The following independent variables were evaluated: the extraction time (*X*_1_: 5, 10 and 15 min), the probe vibration amplitude (*X*_2_: 45, 75 and 95%, the percent expressed is relative measure microns peak to peak -µpp-of the probe, being 100% equivalent to 95.6 µpp), and the solid/solvent ratio (*X*_3_: 5, 10 and 15 g/100 mL of solvent) (Table 1). The predicted data for the response are listed as follows: (i) total content of methylxanthine (caffeine plus theobromine) and (ii) epicatechin, which were obtained using the following model with a generalized second-order polynomial (Equation (1)):

(1) $$Y=\;\beta_{0}+\sum_{i=1}^{k}\beta_{i}X_{i}+\sum_{i}^{k}\beta_{ii}X_{i}^{2}+\;\sum_{i}^{k-1}\sum_{j}^{k}\beta_{ij}X_{i}X_{j}\;$$

where $\;\beta_{0}$, $\beta_{i}$,$\;\beta_{ii}$ and $\beta_{ij}\;$ correspond to the regression coefficients for the intercept term, linear term, quadratic term and interaction term, while *k* is the number of factors tested (*k* = 3) and $X_{i}\;$ and $X_{j}$ are encrypted values of the independent variables.

### 4.7. Statistical Analysis

Data from the experiments are expressed as the mean ± standard deviation (SD). One-way analysis of variance with a least significant difference and Dunnett and Bonferroni multiple comparison tests were used. A *p*-value < 0.05 denoted statistical significance. Analyses were developed using Prism 5 Software from GraphPad Inc. (San Diego, CA, USA).

## 5. Conclusions

In summary, second-quality cocoa beans (SQ) can be considered promising sources of anti-collagenase compounds of interest for the development of anti-aging cosmetic products and promising sources of polyphenols, while cocoa bean shells (BS) can be considered promising sources of methylxanthines. This last cocoa byproduct was selected to analyze the operational parameters of extraction, including extraction time, ultrasound amplitude and solid/solvent concentration using response surface methodology (RSM), because it has the highest value of methylxanthines, a medium concentration of epicatechin and excellent availability and manageability as an agro-industrial residue. The extraction process developed under optimized conditions would obtain almost 2 g and 0.2 g of total xanthines and epicatechin per minute, respectively. In this way, this work contributes to the sustainability and valorization of the cocoa agribusiness, which is an ecological way of exploiting cocoa byproducts from both an economic and environmental standpoint. However, this supports the need to examine the new ingredients from these cocoa byproducts more extensively in in vivo models. Additional efforts to integrate industry and society are also necessary for the development of innovative and sustainable ingredients from cocoa agribusiness.

## Figures and Tables

**Table 1:** Catechins and methylxanthines contents measured by HPLC-DAD-FLD.

Sample	Theobromine (mg/100 g of Sample)	Catechin (mg/100 g of Sample)	Caffeine (mg/100 g of Sample)	Epicatechin (mg/100 g of Sample)
PR	834.7 ± 29.8	17.21 ± 7.68	141.7 ± 10.0 ^ns^	383.1 ± 190.0
CO	855.2 ± 35.4 ^ns^	14.19 ± 3.80 ^ns^	147.0 ± 8.38 ^ns^	279.5 ± 112.0 ^ns^
PA	785.1 ± 31.3 ^ns^	4.62 ± 5.08 **	147.6 ± 34.7 ^ns^	143.9 ± 65.3 **
MC	955.1 ± 28.4 *	10.17 ± 0.15 ^ns^	136.0 ± 3.13 ^ns^	182.5 ± 4.3 ^ns^
SQ	779.6 ± 106 ^ns^	18.15 ± 5.82 ^ns^	138.5 ± 23.8	229.8 ± 51.5 ^ns^
PH	50.3 ± 1.53 ***	0.00 ± 0.00 ***	34.0 ± 5.00 ***	0.0 ± 0.0 ***
BS	1085.0 ± 37.6 ***	16.18 ± 0.13 **	267.0 ± 9.07 ***	104.7 ± 4.2 **

Superscripts indicate significant differences (*p* < 0.05) between cocoa samples respect the control PR by Dunnett’s multiple comparison test; ^ns^ no significative; * *p* < 0.05, ** *p* < 0.01 and *** *p* < 0.001.

**Table 2:** ANOVA results on the extraction of methylxanthines and epicatechin from cocoa bean shells (BS).

Source	Total Xanthines (Caffeine + Theobromine)			Epicatechin		
	Sum of Squares	Degrees of Freedom	*p*-Value	Sum of Squares	Degrees of Freedom	*p*-Value
*X*_1_	110,185	1	0.04	1728	1	0.04
*X*_2_	40,110	1	0.19	103	1	0.55
*X*_3_	575,185	1	0.00	4360	1	0.00
*X*_1_^2^	Excluded			247	1	0.37
*X*_1_ *X*_2_	13,262	1	0.43	Excluded		
*X*_1_ *X*_3_	Excluded			Excluded		
*X*_2_^2^	43,830	1	0.17	656	1	0.16
*X*_2_ *X*_3_	Excluded			53	1	0.67
*X*_3_^2^	55,794	1	0.13	967	1	0.10
Pure error	153,595	8		1864	7	
Cor Total	985,371	15		9756	14	
R^2^	0.85			0.81		

**Table 3:** Experimental measured and predicted values of the independent variables, for different points in the experimental design.

Design Point	Independent Variables			(mg/100 g of Sample)			
	*X*_1_ (min)	*X*_2_ (%)	*X*_3_ (mg/100 mL)	Total Xanthine		Epicatechin	
				Measured	Predicted	Measured	Predicted
1	15	70	5	2622, 76	2816, 55	294, 22	313, 108
2	5	70	15	2191, 30	1992, 99	253, 35	234, 767
3	15	45	10	2592, 62	2521, 26	294, 80	288, 589
4	10	70	10	2275, 96	2253, 91	252, 91	249, 580
5	5	95	10	2316, 08	2375, 59	257, 91	264,121
6	5	70	5	2513, 96	2581, 84	274, 26	283, 710
7	10	70	10	2169, 84	2253, 91	239, 34	249, 580
8	5	45	10	2124, 17	2171, 38	245, 73	248, 652
9	10	70	10	2292, 21	2253, 91	256, 49	249, 580
10	10	95	15	2242, 74	2379, 28	252, 69	265, 214
11	10	45	15	1966, 45	2115, 29	232, 72	248, 534
12	15	95	10	2554, 21	2495, 15	285, 90	282, 978
13	10	95	5	2930, 17	2793, 18	318, 22	302, 406
14	15	70	15	2314, 79	2227, 71	273, 92	264, 165
15	10	45	5	3003, 76	2879, 07	321, 75	309, 228

## Data Availability

Not applicable.

## Supplementary Materials

The following is available online, Table S1: Pearson’s linear correlations and their significance among total polyphenolic compounds (TPC) and antioxidant activity.

## Sample Availability

Samples of the compounds and extracts are available from the authors.

## References

[1] ReportLinker (2020). Global Biomass Power Generation Market 2020–2024. https://www.reportlinker.com/p02255144/Global-Biomass-Power-Generation-Market.html.

[2] Vásquez Z.S., de Carvalho D.P., Pereira G.V.M., Vandenberghe L.P.S., de Oliveira P.Z., Tiburcio P.B., Rogez H.L.G., Neto A.G., Soccol C.R. (2019). Biotechnological approaches for cocoa waste management: A review. Waste Manag..

[3] Shahbandeh M. (2021). Global Cocoa Production 1980–2021; Statista. https://www.statista.com/statistics/262620/global-cocoa-production/.

[4] Okiyama D.C.G., Navarro S.L.B., Rodrigues C.E.C. (2017). Cocoa shell and its compounds: Applications in the food industry. Trends Food Sci. Technol..

[5] Kumar S., Sable K. (2019). Cocoa Products Market; Allied Market Reserarch. https://www.alliedmarketresearch.com/cocoa-products-market.

[6] Ministerio de Agricultura y Desarrollo Rural (2019). Agronet. Reporte: Área, Producción y Rendimiento Nacional Por Cultivo. Bogotá, Colombia. https://www.agronet.gov.co/estadistica/paginas/home.aspx?cod=1.

[7] Nieto K.H., Mendoza N.V., Campos-Vega R., Campos-Vega R., Oomah D., Vergara-Castañeda H.A. (2020). Cocoa by-products. Food Wastes and by-Products: Nutraceutical and Health Potential.

[8] Fedecacao, Federación Nacional de Cacaoteros (2004). El Beneficio y Características Físico Químicas del Cacao (Theobroma cacao L.).

[9] Martín M.Á., Ramos S. (2016). Cocoa polyphenols in oxidative stress: Potential health implications. J. Funct. Foods.

[10] Martín M.Á., Ramos S. (2017). Health beneficial effects of cocoa phenolic compounds: A mini-review. Curr. Opin. Food Sci..

[11] Sharma R., Watson R.R., Preedy V.R., Zibadi S. (2014). Chapter 59-Polyphenols in health and disease: Practice and mechanisms of benefits. Polyphenols in Human Health and Disease.

[12] Wang Y., Feltham B.A., Suh M., Jones P.J.H. (2019). Cocoa flavanols and blood pressure reduction: Is there enough evidence to support a health claim in the United States?. Trends Food Sci. Technol..

[13] Jeon S.E., Choi-Kwon S., Park K.A., Lee H.J., Park M.S., Lee J.H., Kwon S.B., Park K.C. (2003). Dietary supplementation of (+)-catechin protects against UVB-induced skin damage by modulating antioxidant enzyme activities. Photodermatol. Photoimmunol. Photomed..

[14] Karim A.A., Azlan A., Ismail A., Hashim P., Abd Gani S.S., Zainudin B.H., Abdullah N.A. (2014). Phenolic composition, antioxidant, anti-wrinkles and tyrosinase inhibitory activities of cocoa pod extract. BMC Complement. Altern Med..

[15] Abdul Karim A., Azlan A., Hashim P., Abd Gani S.S., Zainudin B.H., Abdullah N.A. (2016). Efficacy of cocoa pod extract as antiwrinkle gel on human skin surface. J. Cosmet. Derm..

[16] Santana Á.L., Macedo G.A. (2018). Health and technological aspects of methylxanthines and polyphenols from Guarana: A review. J. Funct. Foods.

[17] Bartella L., Donna L.D., Napoli A., Siciliano C., Sindona G., Mazzotti F. (2019). A rapid method for the assay of methylxanthines alkaloids: Theobromine, theophylline and caffeine, in cocoa products and drugs by paper spray tandem mass spectrometry. Food Chem..

[18] Carrillo-Hormaza L., Duque L., López-Parra S., Osorio E. (2020). High-intensity ultrasound-assisted extraction of *Garcinia madruno* biflavonoids: Mechanism, kinetics, and productivity. Biochem. Eng. J..

[19] Carrillo-Hormaza L., Ramírez A.M., Osorio E., Gil A. (2017). Optimization of ultrasound-assisted extraction and rapid resolution analysis of flavanols and methylxanthines for the quality control of cocoa-derived products. Food Anal. Methods.

[20] Jiménez N., Carrillo-Hormaza L., Pujol A., Álzate F., Osorio E., Lara-Guzman O. (2015). Antioxidant capacity and phenolic content of commonly used anti-inflammatory medicinal plants in Colombia. Ind. Crops Prod..

[21] Grillo G., Boffa L., Binello A., Mantegna S., Cravotto G., Chemat F., Dizhbite T., Lauberte L., Telysheva G. (2019). Analytical dataset of Ecuadorian cocoa shells and beans. Data Br..

[22] Rodríguez-Carrasco Y., Gaspari A., Graziani G., Santini A., Ritieni A. (2018). Fast analysis of polyphenols and alkaloids in cocoa-based products by ultra-high performance liquid chromatography and Orbitrap high resolution mass spectrometry (UHPLC-Q-Orbitrap-MS/MS). Food Res. Int..

[23] Zhong J.L., Muhammad N., Gu Y.C., Yan W.D. (2019). A simple and efficient method for enrichment of cocoa polyphenols from cocoa bean husks with macroporous resins following a scale-up separation. J. Food Eng..

[24] Natsume M. (2018). Polyphenols: Inflammation. Curr. Pharm. Des..

[25] Qu Z., Liu A., Li P., Liu C., Xiao W., Huang J., Liu Z., Zhang S. (2021). Advances in physiological functions and mechanisms of (−)-epicatechin. Crit. Rev. Food Sci. Nutr..

[26] Gaspar D.P., Chagas Junior G., de Aguiar Andrade E.H., Nascimento L., Chisté R.C., Ferreira N.R., Martins L., Lopes A.S. (2021). How climatic seasons of the Amazon biome affect the aromatic and bioactive profiles of fermented and dried cocoa beans?. Molecules.

[27] Quelal-Vásconez M.A., Lerma-García M.J., Pérez-Esteve E., Arnau-Bonachera A., Barat J.M., Talens P. (2020). Changes in methylxanthines and flavanols during cocoa powder processing and their quantification by near-infrared spectroscopy. LWT.

[28] Carrageta D.F., Dias T.R., Alves M.G., Oliveira P.F., Monteiro M.P., Silva B.M. (2018). Anti-obesity potential of natural methylxanthines. J. Funct. Foods.

[29] Baggott M.J., Childs E., Hart A.B., de Bruin E., Palmer A.A., Wilkinson J.E., de Wit H. (2013). Psychopharmacology of theobromine in healthy volunteers. Psychopharmacology.

[30] Iaia N., Rossin D., Sottero B., Venezia I., Poli G., Biasi F. (2020). Efficacy of theobromine in preventing intestinal CaCo-2 cell damage induced by oxysterols. Arch. Biochem. Biophys..

[31] Arman K. (2012). Theobromine for Tooth Decay Prevention. Cosmet. Toilet.

[32] European Commission (2020). Cosmetic Ingredient Database. https://ec.europa.eu/growth/sectors/cosmetics/cosmetic-ingredient-database_en.

[33] Duque L., Bravo K., Osorio E. (2017). A holistic anti-aging approach applied in selected cultivated medicinal plants: A view of photoprotection of the skin by different mechanisms. Ind. Crops Prod..

[34] Shi M., Nie Y., Zheng X.Q., Lu J.L., Liang Y.R., Ye J.H. (2016). Ultraviolet B (UVB) photosensitivities of tea catechins and the relevant chemical conversions. Molecules.

[35] Arct J., Bielenda B., Oborska A., Pytkowska K. (2003). The tea and its cosmetic aplication. J. Appl. Cosmetol..

[36] Basu-Modak S., Gordon M.J., Dobson L.H., Spencer J.P.E., Rice-Evans C., Tyrrell R.M. (2003). Epicatechin and its methylated metabolite attenuate UVA-induced oxidative damage to human skin fibroblasts. Free Radic. Biol. Med..

[37] Bravo K., Alzate F., Osorio E. (2016). Fruits of selected wild and cultivated andean plants as sources of potential compounds with antioxidant and anti-aging activity. Ind. Crops Prod..

[38] Carrillo L.C., Londoño-Londoño J., Gil A. (2014). Comparison of polyphenol, methylxanthines and antioxidant activity in *Theobroma cacao* beans from different cocoa-growing areas in Colombia. Food Res. Int..

