# Supplementary material for: Chemical and Skincare Property Characterization of the Main Cocoa Byproducts: Extraction Optimization by RSM Approach for Development of Sustainable Ingredients
Source: Molecules. 2021 Dec 7;26(24):7429. doi: 10.3390/molecules26247429 (PMC8709444; doi:10.3390/molecules26247429)
Supplement: Supplementary file 1 [file molecules-26-07429-s001.zip › molecules-1406121-supplementary.pdf]

molecules

Article

Chemical and skincare property characterization of the main cocoa byproducts: extraction optimization by RSM approach for development of sustainable ingredients

Catalina Agudelo [1], Karent Bravo [1], Ana Ramírez-Atehortúa [1], David Torres [1], Luis Carrillo-Hormaza [1] and Edison Osorio [1,*]

[1] Grupo de Investigación en Sustancias Bioactivas, Facultad de Ciencias Farmacéuticas y Alimentarias, Universidad de Antioquia, Calle 70 No. 52-21, Medellín, Colombia; catalina.agudelor@udea.edu.co (CA); karen.bravo@udea.edu.co (KB); anilla0122@gmail.com (ARA); david.torresb@udea.edu.co (DT); luis.carrillo@udea.edu.co (LCC)

* Correspondence: edison.osorio@udea.edu.co; Tel.: +57 6042196590

1. Supplementary Material

Table S1. Pearson's linear correlations and their significance among total polyphenolic compounds (TPC) and antioxidant activity.

Assays	Correlation coefficients
FRAP and TPC	0.993*
ORAC and TPC	0.784*

*P<0.05
